# MyoD phosphorylation on multiple C terminal sites regulates myogenic conversion activity

**DOI:** 10.1016/j.bbrc.2016.11.009

**Published:** 2016-12-02

**Authors:** Laura J.A. Hardwick, John D. Davies, Anna Philpott

**Affiliations:** a Department of Oncology, University of Cambridge, Hutchison/MRC Research Centre, Cambridge Biomedical Campus, Cambridge CB2 0XZ, UK; b Wellcome Trust Centre for Stem Cell Research, University of Cambridge, Tennis Court Road, Cambridge, CB2 1QR, UK; c Peterhouse, University of Cambridge, Trumpington Street, Cambridge, CB2 1RD, UK

**Keywords:** MyoD, Myogenesis, Phosphorylation, bHLH, *Xenopus*, Reprogramming, bHLH, basic-Helix-Loop-Helix, cdk, cyclin-dependent-kinase, ISH, In situ hybridisation, MRF, Muscle Regulatory Factor, SP, serine-proline, TP, threonine-proline, WT, wild-type

## Abstract

MyoD is a master regulator of myogenesis with a potent ability to redirect the cell fate of even terminally differentiated cells. Hence, enhancing the activity of MyoD is an important step to maximising its potential utility for *in vitro* disease modelling and cell replacement therapies. We have previously shown that the reprogramming activity of several neurogenic bHLH proteins can be substantially enhanced by inhibiting their multi-site phosphorylation by proline-directed kinases. Here we have used *Xenopus* embryos as an *in vivo* developmental and reprogramming system to investigate the multi-site phospho-regulation of MyoD during muscle differentiation. We show that, in addition to modification of a previously well-characterised site, Serine 200, MyoD is phosphorylated on multiple additional serine/threonine sites during primary myogenesis. Through mutational analysis, we derive an optimally active phospho-mutant form of MyoD that has a dramatically enhanced ability to drive myogenic reprogramming *in vivo*. Mechanistically, this is achieved through increased protein stability and enhanced chromatin association. Therefore, multi-site phospho-regulation of class II bHLH proteins is conserved across cell lineages and germ layers, and manipulation of phosphorylation of these key regulators may have further potential for enhancing mammalian cell reprogramming.

## Highlights

• MyoD is phosphorylated on multiple S/T-P sites in muscle differentiation.
• Myogenic activity of MyoD is regulated by up to five C terminal phospho-sites.
• Our optimal phospho-mutant form of MyoD dramatically enhances myogenesis *in vivo*.
• MyoD multi-site phosphorylation controls protein stability and chromatin association.
• Multi-site phospho-regulation is a conserved mechanism across cell lineages.

## 1 Introduction

The basic-Helix-Loop-Helix (bHLH) protein MyoD is an extensively studied Muscle Regulatory Factor (MRF) that plays a pivotal role during muscle development, and has also received much attention for its ability to direct a myogenic program even in terminally differentiated somatic cells [1], [2]. Using MyoD to direct trans-differentiation has potential utility in cellular reprogramming efforts directed at *in vitro* modelling of muscle diseases for drug screening [3] and in cell replacement therapies [4]. However, refining of these reprogramming methods requires optimising the myogenic activity of MyoD.

We have previously studied other master regulatory proneural bHLH transcription factors that drive differentiation in the neural lineage, namely Neurogenin2, Ascl1 and NeuroD4 [5], [6], [7], [8]. We see that phosphorylation of these factors on multiple serine-proline and threonine-proline (SP and TP) sites suppressed both their ability to drive neuronal differentiation during development [5], [7] and during cellular reprogramming of mammalian cells [6].

MyoD phosphorylation on Serine 200 (S200) and S5, sites both found as part of an SP motif, has long been recognised to underlie the characteristic MyoD protein fluctuations and activity that vary with cell cycle phase in proliferating myoblasts [9], [10], [11], [12], [13]. MyoD also contains multiple additional SP and TP sites which may, by analogy with proneural factors, play an additional role in regulation of the ability of MyoD to promote myogenic conversion and differentiation. Here, using the *Xenopus* tadpole as a model to test MyoD's myogenic conversion potential, we show that myogenic activity of MyoD is regulated by up to five S/TP kinase sites specifically located in the C terminus of the protein. Furthermore, we see that multi-site phosphorylation regulates both MyoD protein stability and chromatin association. Finally, we identify an optimal phospho-mutant form of MyoD that dramatically enhances myogenic conversion of embryonic ectoderm *in vivo*.

## 2 Materials and methods

### 2.1 Cloning

Wild-type (WT) mouse MyoD in pCS2 has been described [14]. A single C terminal HA tag was added by PCR using the primers: 5′GATCGGATCCACCATGGAGCTTCTATCGCCG-3′; 5′GATCCTCGAGTCAAGCGTAATCTGGAACATCGTATGGGTAAAGCACCTGATAAATCGCATT-3′. All single or multiple site mutations were performed using the QuikChange II or QuikChange Multi Site-Directed Mutagenesis Kit (Agilent Technologies) respectively (PCR primers available on request). Nucleotide and protein sequence alignments were conducted using ClustalW software [15].

### 2.2 *Xenopus laevis* embryo manipulation

Acquisition of *Xenopus laevis* embryos, preparation and injection of synthetic mRNA, staging of embryos and in situ hybridisation (ISH) were conducted as described previously [6], [16], [17]. Dig-oxigenin-labelled anti-sense probes were synthesised from linearised plasmid Actc1 in pSP64T (Xenbase; EcoR1, SP6). Semi-quantitative scoring was conducted for Muscle Actin staining on the injected side of the embryo relative to the uninjected side; grades were assigned 0–3, where 0 indicates no increase in myogenesis, through to 3 indicating extensive myogenesis.

### 2.3 Quantitative real-time PCR (qRT-PCR)

Whole embryo RNA was extracted using the Qiagen RNeasy^®^ Mini Kit and cDNAs were prepared with the QuantiTect^®^ Reverse Transcription Kit (Qiagen). qPCR analysis was conducted using the Quantifast^®^ SYBR Green PCR Kit (Qiagen) in a LightCycler^®^ 480 (Roche). Thermal cycling parameters are described in Ref. [5], and primer sequences (5′–3′): EF1αF = CACCATGAAGCCCTTACTGAG; EF1αR = TGATAACCTGTGCGGTAAATG; xMyh4F = GAACAAGGACCCACTGAACG; xMyh4R = TCCACCTTTACCAGCAGCAT.

### 2.4 Western blotting

Protein extracts were prepared from whole embryos and incubated with or without lambda protein phosphatase (NEB) as described [7], prior to separation on an 18% Tris/Glycine/SDS gel by standard methods. For assay of chromatin association, cross linking, cytoplasmic and chromatin fractionation, western blot and protein quantification were conducted as described [7].

### 2.5 Statistical analysis

For western blotting, experiments were performed in independent duplicate with representative results shown. For qPCR data, mRNA expression was normalised to expression of the housekeeping gene *Elongation Factor 1α (EF1α)*, and for analysis, mRNA levels in injected embryos were calculated relative to stage-matched uninjected controls. Mean values and the standard error of the mean (s.e.m.) were calculated from at least three independent experiments (n = 3). Statistical significance was determined using a paired two-tailed Student's t-test with (p < 0.05) = *; (p < 0.025) = **; (p < 0.0125) = ***. For in situ hybridisation data, experiments were conducted in independent duplicate or triplicate and the n numbers reported refer to the range of total numbers of embryos in each injection category. Statistical significance was determined between categories using a Fisher's Exact Test for Count Data, and p values are as described above.

## 3 Results

### 3.1 MyoD is phosphorylated on sites in addition to S200 during *Xenopus* primary myogenesis

Early studies investigating phospho-regulation of MyoD largely focused on its modification in proliferating cells in culture; either C2C12 myoblasts or C3H10T1/2 fibroblasts transfected with MyoD [10], [12]. To investigate phosphorylation of MyoD and its reprogramming activity *in vivo*, we chose the highly versatile *Xenopus* embryo system, which can be used to study mammalian MyoD activity both in the endogenous myotome and also for reprogramming activity in the non-myogenic ectoderm tissue [14]. Furthermore, the first cleavage of the fertilised egg bisects the embryos into future left and right sides, so unilateral mRNA injection allows MyoD activity to be directly compared between injected and uninjected embryo sides.

**Fig. 1:**
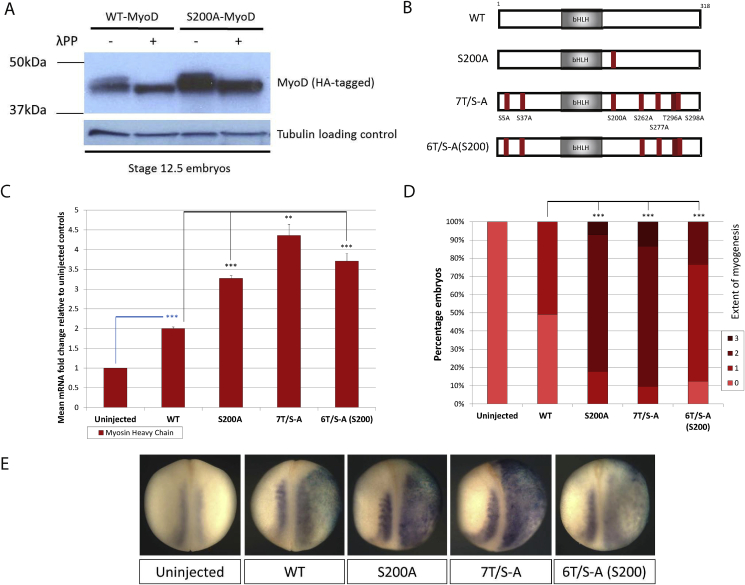
MyoD is phospho-regulated on sites in addition to S200 during primary myogenesis. (A) Western blot analysis of protein extracts from stage 12.5 embryos injected with 200 pg mRNA encoding HA-tagged WT or S200A-MyoD. Samples were incubated with or without lambda protein phosphatase. Tubulin provided a loading control. (B) Schematic representation of WT mouse MyoD protein and phospho-mutant variants, showing approximate locations of SP/TP sites that are mutated to AP in each. (C–E) Embryos were unilaterally injected at the 2-cell stage with 100 pg mRNA encoding the respective MyoD constructs. At stage 18, embryos were assayed by RT-qPCR for Myosin Heavy Chain expression (C [n = 3]) or by ISH with semi-quantitative scoring for Muscle Actin expression (D [n = 52–73]) as described in the methods. Representative embryos are shown in (E); injected side to the right. * = (p < 0.05); ** = (p < 0.025); *** = (p < 0.0125).

Firstly, we explored whether MyoD is phosphorylated during primary myogenesis. Extracts were prepared from stage 12.5 embryos injected with mRNA encoding HA-tagged Wild-Type (WT) MyoD, or S200A MyoD, a mutant version with S200 mutated to alanine to prevent phosphorylation at this established site. Western blot analysis reveals a broad protein band for both WT and S200A proteins (Fig. 1A), with more S200A protein than WT, consistent with previous reports that demonstrate a longer half-life for this mutant protein [10], [11], [12]. Incubation with a broad spectrum protein phosphatase enhances the migration of both WT and S200A, indicating phosphorylation on at least one additional site during differentiation *in vivo*.

### 3.2 A multi-site phospho-mutant MyoD promotes enhanced myogenesis *in vivo*

Mouse MyoD protein contains seven potential proline-directed kinase sites (six serine-proline (SP), and one threonine-proline (TP)) that are highly conserved with human MyoD (Supplementary Fig. 1). To further explore a potential regulatory role of S/TP sites additional to S200 during myogenesis, a panel of phospho-mutant forms of MyoD were created (Fig. 1B): 7T/S-A MyoD contains all seven SP/TP sites mutated to alanine-proline (AP), while 6T/S-A(S200) has the S200 site restored but the remaining six sites mutated. In order to compare their myogenic activity, mRNA encoding these proteins was injected unilaterally into two-cell stage *Xenopus* embryos, and myogenesis was assayed in stage 18 embryos by RT-qPCR and in situ hybridisation (ISH) for muscle structural genes Myosin Heavy Chain or Muscle Actin respectively (Fig. 1C–E). At this level of over-expression, WT MyoD induces a small increase in myogenesis, largely confined to expansion of the myotome on the injected side. By comparison, both S200A and full phospho-mutant 7T/S-A induce a marked increase in myogenesis spreading laterally, and demonstrating reprogramming of ectoderm tissue. Interestingly, 6T/S-A(S200) also induces a significant increase in myogenesis relative to WT MyoD, albeit with less activity than S200A or 7T/S-A MyoDs. This demonstrates that sites in addition to S200 can regulate myogenic activity of MyoD.

### 3.3 Regulatory activity resides in C terminal phosphorylation sites

**Fig. 2:**
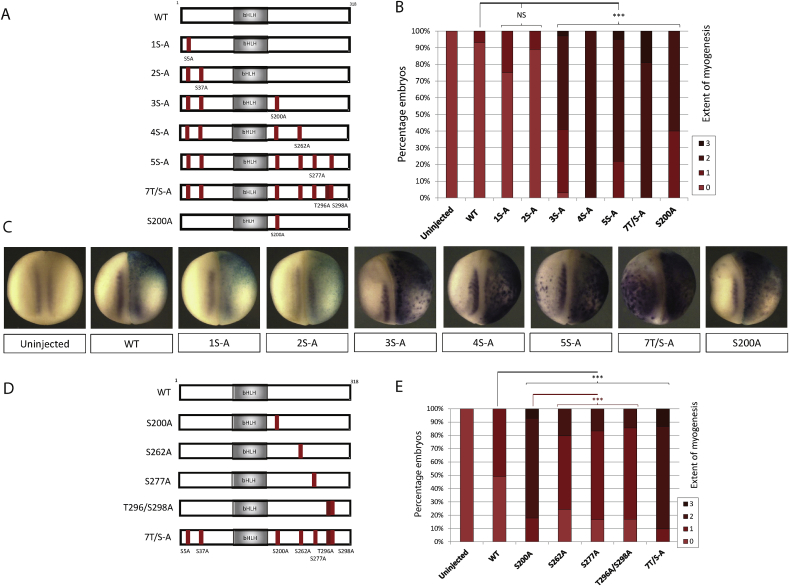
Mutational analysis shows regulatory activity of S200 and additional C terminal phosphorylation sites. Schematic representation of phospho-mutant constructs with mutation of cumulative (A) or individual (D) sites. Two-cell stage embryos were unilaterally injected with 100 pg mRNA of the respective MyoD construct and assayed at stage 18 for expression of Muscle Actin by ISH: (B) Cumulative mutant series [n = 68–74] with representative images shown in (C). (D) Single site mutants [n = 52–83]. NS = Not significant; * = (p < 0.05); ** = (p < 0.025); *** = (p < 0.0125).

We have previously described a model for phospho-regulation of proneural protein activity, where the number, rather than location of available phospho-sites is the key determinant of bHLH activity; mutation of increasing numbers of phosphorylation sites additively increases the neurogenic activity of the mutant proteins [5], [7]. To explore if a similar phenomenon may control regulation of MyoD, a panel of MyoD phospho-mutants were made with SP/TP sites cumulatively mutated from the N terminus to generate 1S-A, 2S-A etc (Fig. 2A). Due to the immediate proximity of T296/S298, these sites were mutated simultaneously.

Embryos were injected with mRNA as before and scored for myogenesis at stage 18 by ISH (Fig. 2B–C). Mutation of the N terminal SP sites in MyoD has no significant effect on its activity relative to the wild-type protein. Similarly, mutation of S200 in addition to the two N terminal sites (i.e. 3S-A) produces a level of myogenesis comparable to mutation of the single S200 site alone, suggesting that the N terminal sites do not contribute to regulation of MyoD myogenic activity. However, mutation of C terminal residues in addition to S200 results in a more active MyoD than the single phosphorylation site mutant S200A MyoD. Thus, for MyoD, both number *and* location of phospho-sites contribute to its regulation.

### 3.4 S200 is the most important individual C terminal site for regulating myogenic activity

The five C terminal phospho-sites in mouse MyoD are also the phospho-sites that are most conserved with both human and *Xenopus* MyoD (Supplementary Fig. 1); S200 and S262 are highly conserved between all three species, suggesting that these may have enhanced functional significance. We therefore sought to address whether myogenic activity depends on any particular C terminal phospho-site.

Each C terminal site was mutated individually and myogenic activity was assayed as described above (Fig. 2D–E). Mutation of any individual C terminal phospho-site results in significantly enhanced myogenesis compared to WT MyoD. Furthermore, the three most C terminal sites when mutated individually (S262, S277 and T296/S298) are very similar to each other in inducing a moderate increase in myogenesis. By comparison, mutation of the S200 site alone results in myogenic activity that is significantly greater than any other single site mutant, yet this is still not as active as the full 7T/S-A phospho-mutant. Therefore, S200 confers the most significant regulatory activity but its mutation alone is not sufficient to maximally activate MyoD; additional C terminal residues must also be mutated.

### 3.5 Identification of an optimal phospho-mutant MyoD with maximal myogenic activity

**Fig. 3:**
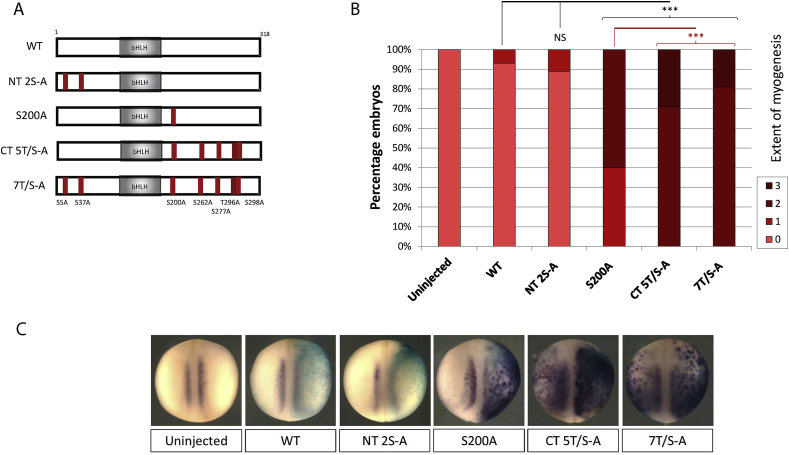
Maximal myogenic activity is achieved by mutation of multiple C terminal sites. (A) Schematic representation of phospho-mutant constructs. (B) Two-cell stage embryos were unilaterally injected with 100 pg mRNA of the respective MyoD construct and assayed at stage 18 as before [n = 32–41] with representative images shown in (C). NS = Not significant; * = (p < 0.05); ** = (p < 0.025); *** = (p < 0.0125).

For the purposes of cellular reprogramming, an optimal form of MyoD would have maximal activity in driving myogenic conversion of non-muscle tissue. Therefore, we finally sought to derive an optimal phospho-mutant MyoD in our *Xenopus* assay. For the proneural proteins, maximal activity is achieved by mutation of all available phospho-sites [5], [6], [7], but for MyoD, we see that N terminal sites do not contribute to regulation (Fig. 2). S200 is the most important single C terminal site (Fig. 2), so additional constructs were made to mutate S200 in combination with other C terminal sites (data not shown). In all assays conducted, the most extensive myogenesis is induced by CT 5T/S-A MyoD, in which all five C terminal phospho-sites are mutated but the two N-terminal phospho-sites are intact. These results are summarised in Fig. 3, where NT 2S-A MyoD shows equivalent activity to WT protein, and CT 5T/S-A MyoD is more active than the full 7T/S-A phospho-mutant. We note that both of these multi-site mutants are significantly more active than the single S200A mutant, inducing extensive conversion of the lateral ectoderm to a myogenic cell fate, often seen bilaterally over the embryo. Thus, while S200 shows the greatest regulatory role of any individual site, phosphorylation must be prevented on all five C-terminal serine/threonines for maximal differentiation activity.

### 3.6 CT 5T/S-A MyoD has both increased protein accumulation and enhanced chromatin binding relative to WT and S200A MyoD

**Fig. 4:**
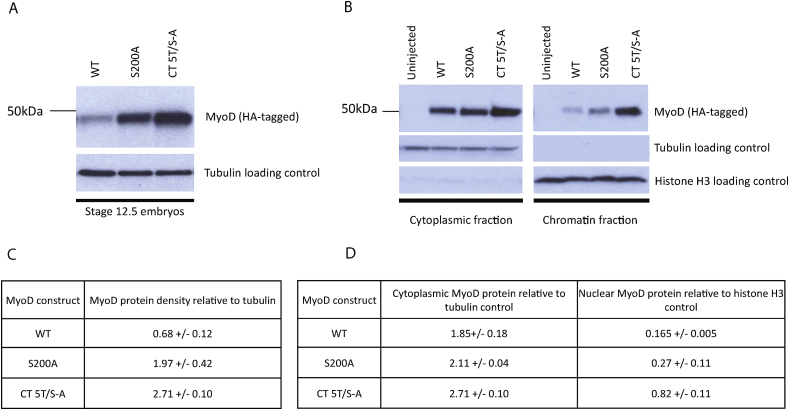
CT 5T/S-A MyoD shows enhanced protein stability and enhanced chromatin binding relative to both WT and S200A MyoD. Embryos were injected with 200 pg mRNA encoding HA-tagged MyoD constructs as indicated, and western blot analysis was performed on whole embryo extracts at stage 12.5 (A) or cross linking and cytoplasmic/chromatin extracts at stage 13 (B). MyoD protein density was quantified relative to tubulin loading control for whole embryo extracts (C) and cytoplasmic fractions (D), or relative to histone H3 for chromatin fractions (D). Mean values are shown from independent duplicate samples with standard error of the mean.

Having established an optimal phospho-mutant form of MyoD that has superior myogenic activity *in vivo*, we investigated the mechanisms by which this enhanced differentiation is achieved. One mechanism may be by enhancing MyoD protein stability. We have already shown that injection of equal amounts of WT and S200A MyoD mRNA produces a greater accumulation of S200A protein relative to WT MyoD (Fig. 1) so we next determined whether the optimal phospho-mutant CT 5T/S-A MyoD showed further enhancement of stability. HA-tagged mRNA was injected into *Xenopus* embryos, and protein extracts were prepared at stage 12.5 for western blot analysis; the density of each MyoD protein band was calculated relative to the respective tubulin loading control (Fig. 4A, C). While injection of the same amount of mRNA results in an almost three-fold increase in S200A protein relative to WT MyoD, CT 5T/S-A MyoD protein accumulates to a level that is more than four-fold higher than WT MyoD. Thus, whilst S200 is an established phospho-site that regulates MyoD stability [10], [12], further enhancement of MyoD protein stability accompanies mutation of additional C-terminal SP sites.

Phospho-mutant proneural proteins show both an enhanced stability compared to their wild-type counterparts, but also exhibit enhanced DNA binding affinity, and both of these attributes contribute to superior reprogramming activity [5], [7]. To test whether MyoD phospho-status influences protein binding to embryonic chromatin, embryos were injected as before, and cross-linking was performed in stage 13 embryos prior to nuclear extraction, chromatin isolation and western blot analysis (Fig. 4B, D). Cytoplasmic samples were also collected and MyoD protein accumulation was calculated relative to tubulin or histone H3 loading controls in cytoplasmic and chromatin fractions respectively. The absence of tubulin protein in the chromatin fraction confirms successful chromatin isolation, and no MyoD protein was detected in the uninjected embryos, confirming specificity of the anti-HA antibody.

Comparing the relative amounts of chromatin bound MyoD protein (Fig. 4D) with the relative amounts of whole embryo MyoD protein (Fig. 4C), S200A MyoD is only 1.6-fold higher than WT MyoD in chromatin samples, suggesting that S200A displays greater protein stability than WT MyoD, but no greater chromatin binding affinity. Therefore, protein stability alone contributes to the enhanced activity of this mutant S200A protein. In contrast, chromatin-bound CT 5T/S-A MyoD accumulates to five-fold higher than WT and three-fold higher than S200A MyoD, demonstrating an increased chromatin binding affinity in addition to increased protein accumulation. Thus, our optimal phospho-mutant CT 5T/S-A dramatically enhances myogenesis *in vivo* by a combination of increased protein accumulation and enhanced chromatin binding; this latter feature being unique to the CT 5T/S-A MyoD phospho-mutant and not shared with S200A MyoD.

## 4 Discussion

The bHLH transcription factor MyoD is a master regulator of myogenesis, and manipulating its activity both for *in vitro* modelling of muscle disease and for cell replacement therapy has huge potential in translation medicine and cellular reprogramming [18]. We set out to engineer a maximally active form of MyoD for *in vivo* reprogramming in the *Xenopus* frog system. Neurogenin2, Ascl1 and NeuroD4, bHLH transcription factors regulating neurogenesis, undergo multi-site phosphorylation that coordinates neuronal differentiation with the cellular kinase environment *in vivo* [5], [6], [7], [8]. As such, multi-site bHLH protein phosphorylation in rapidly dividing cells suppresses their ability to drive neuronal differentiation; a multiply phospho-mutant form of these proneural proteins substantially enhances their ability to reprogram both *Xenopus* ectoderm and mammalian fibroblasts into neurons [5], [6], [7], [8]. Here, we show here that multi-site phosphorylation also controls the ability of MyoD to regulate cellular reprogramming to muscle *in vivo*, demonstrating conservation of a similar regulation of bHLH proteins across cell lineages and germ layers.

Analogous to our findings for proneural bHLH proteins [5], [6], [7], [8], we see that MyoD is phospho-regulated on multiple proline-directed kinase sites during developmental myogenesis. Moreover, by combining mutational analysis with this *in vivo* assay system, we have identified an optimal phospho-mutant form of MyoD, CT 5T/S-A MyoD, that displays markedly enhanced ability to drive myogenic differentiation compared to any previously described phospho-mutant [9], [10], [11], [12], [13]. Our optimal C terminal phospho-mutant protein displays increased protein stability relative to both WT MyoD and S200A MyoD, a mutant previously shown to have enhanced stability and activity compared to the WT protein [10], [11], [12]. In addition, we also see that CT 5T/S-A MyoD has increased chromatin affinity that is not shared by S200A MyoD. The data we present here, in combination with previous work on proneural proteins [5], [6], [7], [8], support the conservation of a phospho-regulatory model of bHLH protein action, both functionally and mechanistically, across both nerve and muscle cell lineages.

However, in contrast to proneural proteins where only the number of available phospho-sites is key to regulating protein function [5], [6], [7], [8], we find that both number *and* location of phospho-sites are important for MyoD, with regulatory phospho-sites located solely in the C terminus of the protein. In contrast to MyoD N terminal phospho-sites, C terminal sites show strong sequence conservation between species. Indeed, even in proliferating myoblasts, four major isoforms of MyoD were found to be subsequently resolved by phosphatase treatment [10], and an early *in vitro* assay found increased reporter transactivation when other individual C terminal proline-directed kinase sites were mutated [12]. These were found not to be phosphorylated by cdks in dividing culture cells, but they may be targeted by other proline-directed kinases such as MAPKs in the environment of the differentiating myotome.

The distinct C terminal location of regulatory phospho-sites may also relate to the complexity of MyoD function that is being revealed by genome-wide analysis. MyoD temporally orchestrates expression of multiple subsets of genes during the specification and differentiation stages of myogenesis [19]. Additionally, MyoD has been found to bind at sites throughout the genome where it induces histone modifications rather than gene expression [20]. Thus, for MyoD, DNA binding affinity does not always correlate with transcriptional activation and instead, the timing and location of cofactor association may be the critical determinant for temporal and spatial activity. For example, genome-wide analysis has shown that MyoD transcriptional activity is temporally regulated through a direct repression of promoter-bound MyoD [19]. A comparison of the genome-wide promoter occupancy of both WT and phospho-mutant MyoD may reveal clues as to potential differential phospho-regulation of distinct downstream MyoD targets. Furthermore, consistent with a role in cofactor association, the ability of MyoD to activate transcription in inaccessible chromatin has been mapped to the C terminal domain [21], and partly attributed to the helix 3 domain [22] that enables binding to pioneer factors Pbx/Meis [23]; spanning resides 218–269, this includes the S262 phospho-site. It will be interesting to determine whether co-factor binding is regulated by MyoD phosphorylation.

In conclusion, we have characterised multi-site phospho-regulation of MyoD on C-terminal S/TP sites that controls its ability to drive myogenesis *in vivo*: Whilst there are differences between the myogenic and neurogenic bHLH factors, the essence of multi-site phospho-regulation is both functionally and mechanistically conserved in muscle and nerve. Using an *in vivo* developmental and reprogramming assay system in *Xenopus* embryos, we have derived a novel C terminal phospho-mutant MyoD protein that has significantly enhanced myogenic activity compared to any previously described mutants. This has the potential for enhancing trans-differentiation of mammalian cells into muscle for translational applications, and this should form the basis of further investigations.

## Appendix A Supplementary data

Supplementary Fig. 1Protein sequence alignment of human, mouse and *Xenopus* MyoD. The bHLH domain is underlined and SP/TP sites are highlighted in red. A consensus line is also shown below the alignment to indicate the degree of conservation at each position: Residues may be identical (*), strongly conserved (:) or weakly conserved (.).
