# Supplementary material for: MyoD phosphorylation on multiple C terminal sites regulates myogenic conversion activity
Source: Biochem Biophys Res Commun. 2016 Dec 2;481(1-2):97–103. doi: 10.1016/j.bbrc.2016.11.009 (PMC5127879; doi:10.1016/j.bbrc.2016.11.009)
Supplement: Supplementary file 1 — Supplementary Fig. 1Protein sequence alignment of human, mouse and Xenopus MyoD. The bHLH domain is underlined and SP/TP sites are highlighted in red. A consensus line is also shown below the alignment to indicate the degree of conservation at each position: Residues may be identical (*), strongly conserved (:) or weakly conserved (.). [file mmc1.pdf]

A

```
hMyoD               MELLSPPLRDVDLTAPDGSLCSFATTDDFYDDPCFDSPDLRFFEDLDPRLMHVGALLKPE
mMyoD               MELLSPPLRDIDLTGPDGSLCSFETADDFYDDPCFDSPDLRFFEDLDPRLVHMGALLKPE
xMyoD1              MELLPPPLRDMEVT--EGSLCAFPTPDDFYDDPCFNTSDMSFFEDLDPRLVHVTLLKPEE
                    ****.*****:::*  :****:* *.*********::.*: *********:*:  *   *

hMyoD               EHSHFPAAVHPAPGAREDEHVRAPSGHHQAGRCLLWACKACKRKTTNADRRKAATMRERR
mMyoD               EHAHFPTAVHPGPGAREDEHVRAPSGHHQAGRCLLWACKACKRKTTNADRRKAATMRERR
xMyoD1              ------------PHHNEDEHVRAPSGHHQAGRCLLWACKACKRKTTNADRRKAATMRERR
                                *  .********************************************

hMyoD               RLSKVNEAFETLKRCTSSNPNQRLPKVEILRNAIRYIEGLQALLRDQD----AAPPGAAA
mMyoD               RLSKVNEAFETLKRCTSSNPNQRLPKVEILRNAIRYIEGLQALLRDQD----AAPP-GAA
xMyoD1              RLSKVNEAFETLKRYTSTNPNQRLPKVEILRNAIRYIESLQSLLHDQDEAFY--------
                    ************** **:********************.**:**:***

hMyoD               AFYAPGPLPPGRGGEHYSGDSDASSPRSNCSDGMMDYSGPPSGARRRNCYEGAYYNEAPS
mMyoD               AFYAPGPLPPGRGSEHYSGDSDASSPRSNCSDGMMDYSGPPSGPRRQNGYDTAYYSEAAR
xMyoD1              -----------PVLEHYSGDSDASSPRSNCSDGMMDYNSPPCGSRRRNSYDSSFYSDSPN
                                  ***********************..**.*.**:* *: ::*.::.

hMyoD               EPRPGKSAAVSSLDCLSSIVERISTESPAAPALLLADVPSESPPRRQEAAAPSEGESSGD
mMyoD               ESRPGKSAAVSSLDCLSSIVERISTDSPAAPALLLADAPPESPPGPPEGASLSDTEQG-T
xMyoD1              DSRLGKSSVISSLDCLSSIVERISTQSPSCPVPTAVDSGSEGSPCSPLQGETLSERVITI
                    :.* ***:.:***************:**:.*.   .*  .*..*     .   . .

hMyoD               PTQSPDAAPQCPAGANPNPIYQVL
mMyoD               QTPSPDAAPQCPAGSNPNAIYQVL
xMyoD1              PSPSNTCTQLSQDPSS--TIYHVL
                     : *  .:  .   :.  .**:**
```

Supplementary Figure 1
